# The physiology of survival: Fluid and food

**DOI:** 10.1113/EP093194

**Published:** 2025-09-02

**Authors:** Mike Tipton, Hugh Montgomery

**Affiliations:** ^1^ Extreme Environments Laboratory, SPSHS University of Portsmouth Portsmouth UK; ^2^ Institute of Sport, Exercise and Health University College London London UK

It is a sad reflection of where we are in 2025 that the most common question currently posed by international media to extreme environmental physiologists is: What is the minimum amount of fluid and food required daily to survive? We offer some information below. We hope that no one finds themselves in a situation where the unavailability of fluid and food becomes life‐threatening.

## 1 FLUID

A number of intrinsic and extrinsic factors will influence the daily loss of water from the body. A resting human in a thermoneutral environment (air temp. 26–28°C, 50% relative humidity) will lose about 500 mL day^−1^ through unavoidable and unregulated transudation of water through the skin (insensible skin loss). More is lost as inspired air is warmed to body temperature, saturated with water vapour and then expired: usually 500 mL day^−1^, but up to 2.4 L day^−1^ in dry hot (desert) or dry cold (polar or at altitude) conditions. Physical work increases fluid loss via this ventilatory route, but also increases body temperature and thus sweat loss – at a rate of up to 2 L h^−1^ (Anderson, 1977). If in a dry environment, the evaporation of this sweat will cool the skin surface, but if in an environment with a high water vapour pressure, the sweat will not evaporate and therefore cool, but will simply drip from the body.

Waste products of metabolism need to be dissolved in water to be excreted via the kidney. Overall, a minimum of about 500 mL day^−1^ of water is lost as urine; however, protein catabolism (in starvation, for instance) can increase this volume. Amino acids from proteolysis (mainly alanine and glutamine) are metabolised to generate carbon dioxide, water and ammonia (the latter being toxic to cells if it accumulates). Ammonia toxicity is prevented by its combination with carbon dioxide in the liver to form urea which, in normal amounts, is not toxic to cells. The urea leaves the liver and is excreted by the kidneys in urine. This requires approximately 50 mL for each gram of urea in the urine (2–3 mL for every gram of protein consumed). Those suffering a shortage of water should avoid a protein‐rich diet, but in starvation conditions, even when not eating any protein, the body uses its own muscle protein as a source of energy (Jequier & Constant, 2009; Mellanby, 1942). For the purposes of estimating water losses in a survival situation, water losses in faeces are relatively small, unless the individual is suffering from diarrhoea (Anderson, 1977).

Water is, of course, gained through ingestion – most as liquid, but 20–30% from ‘wet food’. Food metabolism also generates ‘metabolic water’ – about 350–500 mL day^−1^ (i.e. about 25–33% of the daily water requirement of a sedentary person), depending on the nature of the diet (i.e. whether or not dehydration is accompanied by starvation). The complete breakdown of 100 g of carbohydrate and lipid produces 55 and 107 g of metabolic water, respectively, and both therefore contribute to body water requirements.

Therefore, in *resting*, *thermoneutral circumstances* (minimum water losses), a person in a survival situation will need around 1.5 L day^−1^, but internal (e.g. exercise) and external factors (e.g. air temperature) can increase this requirement significantly. In a survival situation at rest, urine losses will be less, as will the water gained from metabolism. Metabolic water production may contribute about 0.5 L day^−1^, but this will depend on food availability and its constituents. The net result is a minimum average requirement to drink in these conditions of around 1 L day^−1^. This volume can easily increase towards 4 L day^−1^ and beyond to balance loss through sweating and respiration when ambulatory in hot conditions (Sawka et al., 2005). It is worth noting that although some ‘survival rations’ provide a smaller volume of water per day (110–220 mL day^−1^ for those at rest in a life raft, for example), this is not a long‐term option for people trying to survive day to day ambulatory living in hot conditions (Hervey & McCance, 1954; McCance et al., 1956).

If the minimum volume for fluid balance is not available, dehydration occurs. Circulating blood volume is initially maintained at the expense of the intracellular and interstitial fluid volumes. The extent of this fluid deficit may be reduced by adopting, where possible, a good water conservation strategy (e.g. avoiding activities and conditions that promote sweating). As noted, factors such as age, health status, climatic conditions and activity level significantly impact on the progression of dehydration.

The effect of the fluid deficit will depend on its magnitude. Dehydration initially results in reduced work capacity, higher deep body temperatures, loss of heat acclimatisation, increased cardiovascular‐related problems and increased likelihood of heat illness. Body fluid losses in excess of about 5% body weight, especially in warm environments, can be associated with headache, irritability and feelings of light‐headedness (Craig & Cummings, 1966). When body fluid losses reach about 8–10% of body weight, performance becomes significantly impaired and there may be weakness, dizziness and faintness; a rapid low‐volume (‘thready’) pulse; and rapid shallow breathing, possibly associated with pins and needles of the fingertips and around the mouth. As dehydration progresses the skin appears to lose elasticity and exposed mucus membranes (mouth, nose, throat) become ‘dry’. After a relatively short space of time without sufficient water, the skin will appear to ‘shrink’ and become shrivelled; the lips almost disappear leaving teeth and gums protruding, while the limbs become shrunken and emaciated (Golden & Tipton, 2002). The concentration of electrolytes in the remaining body fluids increases above normal levels (e.g. high sodium concentration [hypernatraemia] and urea concentration), resulting in a hyperosmolar state, intracellular dehydration and disturbed cellular function. This may be responsible for many of the undesirable side effects of dehydration, including intense thirst, delirium and hallucinations. Severe dehydration results in hypotension, circulatory collapse and organ failure. Death usually occurs with acute losses in the range of 15–20% body water. This can occur in as little as 3 days in hot climates with no access to water (Wolf, 1958).

*After a week, the terrible thirst became a bigger problem than the general discomfort and intense heat from the sun. It was no longer simply a question of a dry mouth; now our tongues were swollen and furred, while our lips were cracked. It was difficult to muster a spit and eating our hard tack (biscuit) was impossible. After a quarter of an hour of chewing we still couldn't swallow it and in the end simply blew the powder away like dust*.

WW II survivor (in Golden & Tipton, 2002)

## 2 FOOD

In comparison with dehydration, starvation is less of an immediate threat and for a hydrated individual to die of starvation requires weeks of inadequate food provision. Therefore, death from starvation is indicative of long‐term food deprivation and should be avoidable in all but extreme survival scenarios.

The average energy expenditure for a human is about 2400 kcal day^−1^ with normal activity, and about 1400 kcal day^−1^ if at rest. Normally, carbohydrate and fat intakes satisfy most of the energy requirements (63% and 25%, respectively), with the relative proportion varying depending on the tissue in question, and the intensity and duration of the activity (DeLany & Lovejoy, 1996; Westerterp, 2017).

The major cause of variation in energy expenditure is exercise, which can increase it up to 25 times that seen at rest for varying durations depending on the activity and its intensity. Normally, however, only 15–33% of a person's total daily energy expenditure is accounted for by obvious physical activity. The rest is required to sustain the body's vital functions (‘basal metabolic rate’), these accounting for about 1 kcal min^−1^ of energy expenditure.

If the intake of metabolic fuels is lower than that expended, the body initially uses its reserves of carbohydrate (first few days). Liver glycogen stores can become depleted in as little as 24 h during starvation at rest with shivering (Tipton et al., 1997). Fat with some carbohydrate is then used (weeks). Finally, protein is used as a metabolic fuel which, if sustained, leads to an increasingly severe loss of muscle mass (circa 0.5 kg day^−1^ in total starvation of an otherwise unstressed adult). This (see above) can drive increased urinary water loss and thus dehydration.

Starvation does evoke some compensatory mechanisms such as a fall in basal metabolic rate (due to loss of muscle mass and decreased activation of the sympathetic nervous system); increased gluconeogenesis early on (which can reduce the rate of protein loss by a factor of 4 over 28 days); and a decline in physical activity. Consequently, the body can survive longer before serious disruption (Cahill, 1978).

Decrements in physical and cognitive performance begin when 10% or more of body weight is lost in well‐hydrated individuals. Fit and healthy individuals appear to be able to maintain normal work capacity during short periods (<10 days) on severely restricted diets. It is difficult to separate the starved from the dehydrated and, as noted, more significant acute problems can occur from small reductions in body weight when the cause of this reduction is dehydration.

If under‐consumption of food continues for a sufficient duration, changes occur in the aerobic capacity of muscle and the oxygen carrying capacity of the blood. Stamina and physical work capacity are also reduced (Pichan et al., 1988). Hypoglycaemia occurs when required to perform light to moderate exercise, and results in fatigue as muscle and liver glycogen stores become severely depleted, blood glucose levels fall, and the ability to metabolise fat is impaired (Marriott, 1995). Aerobic capacity appears unaffected up to a 10% reduction in body weight and thereafter is reduced by 4% for every 1% reduction in weight. These changes are caused, in part, by a reduction in the mass of metabolically active tissue. Loss of muscle also changes muscle biochemistry and reduces strength. Studies have reported a 21–24% reduction in maximal lifting capability during 8 weeks of reduced food intake, when body weight fell by 13–16% (Golden & Tipton, 2002; Weiss et al., 2017). In contrast to these decrements, grip strength appears to be relatively well maintained (Johnson et al., 1994).

The loss of significant (∼20%) lean tissue is associated with damage to the heart, lungs, kidneys and liver; a compromised immune system – possibly due to the reduction in the concentration of the amino acids associated with immune function – and resulting reduction in resistance to infection and delayed recovery from injury; electrolyte imbalances, which, along with negative structural changes to the heart, result in cardiac arrythmias, bradycardia, hypotension and heart failure; hormonal imbalances, which impact metabolism, bone health and reproductive function; impaired thermoregulation; ‘starvation diarrhoea’ due to impaired gut endothelial integrity and function; and disturbances to vision and hearing. Over time, micronutrient deficiency (lack of vitamins and minerals or ‘hidden hunger’) can result in a wide range of problems including anaemia, problems with pregnancy, stunted growth, blindness, and impaired immune and cognitive function (Ritchie & Roser, 2017).

In the absence of mental or physical stress, body weight losses of 6% or less over periods of 10–45 days produce no degradation in cognitive performance as defined by tests of intellectual behaviour (e.g. memory, reasoning, decision making, vigilance, reaction time). In the classic study of Keys et al. (1950), which was designed to induce a slow, steady and eventually a severe loss of weight, little indication of changes in the cognitive performance of the group was observed, although individual participants did report cognitive decline, memory lapses, inability to concentrate, confusion, obsessive behaviours (e.g. with food), apathy, lethargy, indifference and social withdrawal. Psychiatric deterioration occurred in 25% of the subjects. Analyses of behaviour during famines, men lost at sea and prisoners of war, show similar findings, and confirm that lethargy, helplessness and hypochondria disrupt cognitive performance. However, they do not prevent intelligent and purposeful behaviour when the opportunity arises to procure food or escape the situation. Studies in which starvation has been combined with other stresses (such as sleep deprivation and danger), suggest that cognitive performance degradation of 5–35% can occur within days (Stahle et al., 2011).

Death in starvation thus results from protein loss and dehydration as well as from loss of essential micronutrients, and is most often mediated by infection (poor immune function), organ failure or cardiac arrest. In complete starvation, this usually occurs within 40–60 days, the precise timing being dependent on the body's ability to conserve protein for as long as possible while maintaining a supply of energy to vital organs, and on the physical condition of the individual on entering into a period of negative energy balance. In lean people, with low reserves, there is a relatively large loss of tissue protein when food intake is inadequate. The elderly are at greater risk and children are also more susceptible to starvation due to their higher nutritional needs for growth and the fact that starvation represents a stressor to their under‐developed immune system, increasing the chances of life‐threatening disease.

It is concluded that fluid and energy balance are intimately related and that their maintenance is critical to physical and mental function, health and survival.

## AUTHOR CONTRIBUTIONS

Both authors have read and approved the final version of this manuscript and agree to be accountable for all aspects of the work in ensuring that questions related to the accuracy or integrity of any part of the work are appropriately investigated and resolved. All persons designated as authors qualify for authorship, and all those who qualify for authorship are listed.

## CONFLICT OF INTEREST

None declared.

## FUNDING INFORMATION

None.
